# Association of ATP binding cassette transporter G8 rs4148217 SNP and serum lipid levels in Mulao and Han nationalities

**DOI:** 10.1186/1476-511X-11-46

**Published:** 2012-05-01

**Authors:** Qing Li, Xian-Liang Wei, Rui-Xing Yin

**Affiliations:** 1 Department of Cardiology, Institute of Cardiovascular Diseases, the First Affiliated Hospital, Guangxi Medical University, 22 Shuangyong Road, Nanning 530021, Guangxi, People’s Republic of China; 2 Department of Anatomy, School of Premedical Sciences, Guangxi Medical University, Nanning, 530021, Guangxi, People’s Republic of China

## Abstract

**Background:**

The association of ATP binding cassette transporter G8 gene (*ABCG8*) rs4148217 single nucleotide polymorphism (SNP) and serum lipid profiles is still controversial in diverse racial/ethnic groups. Mulao nationality is an isolated minority in China. The aim of this study was to evaluate the association of *ABCG8* rs4148217 SNP and several environmental factors with serum lipid levels in the Guangxi Mulao and Han populations.

**Methods:**

A total of 634 subjects of Mulao nationality and 717 participants of Han nationality were randomly selected from our previous samples. Genotyping of the *ABCG8* rs4148217 SNP was performed by polymerase chain reaction and restriction fragment length polymorphism combined with gel electrophoresis, and then confirmed by direct sequencing.

**Results:**

The genotypic and allelic frequencies of *ABCG8* rs4148217 SNP were different between the two nationalities (*P* < 0.01 for each), the frequency of A allele was higher in Mulao than in Han. The A allele carriers in Han had lower high-density lipoprotein cholesterol (HDL-C) and apolipoprotein (Apo) A1 levels than the A allele noncarriers (*P* < 0.05 for each), whereas the A allele carriers in Mulao had lower ApoA1 levels than the A allele noncarriers (*P* < 0.05). Subgroup analyses showed that the A allele carriers in Han had lower HDL-C and higher triglyceride (TG) levels in females but not in males than the A allele noncarriers (*P* < 0.05 for each), and the A allele carriers in Mulao had lower ApoA1 levels in females but not in males than the A allele noncarriers (*P* < 0.05). The levels of TG and HDL-C in Han, and ApoA1 in Mulao were associated with genotypes in females but not in males (*P* < 0.05-0.01). Serum lipid parameters were also correlated with several environmental factors (*P* < 0.05-0.001).

**Conclusions:**

The *ABCG8* rs4148217 SNP is associated with serum TG, HDL-C and ApoA1 levels in our study populations, but this association is different between the Mulao and Han populations. There is a sex (female)-specific association in both ethnic groups.

## Introduction

Coronary heart disease (CHD) continues to be the leading cause of morbidity and mortality among adults nowadays [1]. Epidemiologic studies enable one to predict most of the potential victims of this disease, years before they become ill. However, this disease formation is mainly caused by dyslipidemia, such as a low concentration of high-density lipoprotein cholesterol (HDL-C) [2,3] and apolipoprotein (Apo) A1 [4,5], and high level of total cholesterol (TC) [6], triglyceride (TG) [7], low-density lipoprotein cholesterol (LDL-C) [8], and ApoB [4,9], and other factors such as hypertension [10], and cigarette smoking [11].

It is widely accepted that dyslipidemia is caused by multiple environmental and genetic factors and their interactions [12]. Previous family and twin studies have proved that the genetic background accounts for almost a half of the total disease risk [13,14]. Linkage and case–control studies of candidate genes and recent genome-wide studies have identified multiple genes leading to dyslipidemia. ATP binding cassette transporter G8 (ABCG8) is cholesterol half-transporter that function together with G5 as a heterodimer [15]. Expression of these transporters mediates the efflux of cholesterol and plant sterols from enterocytes back into the intestinal lumen and their excretion into the bile, thus limiting their accumulation in the body and promoting reverse cholesterol transport (RCT) [16,17]. In humans, deleterious mutations in either of these genes cause the genetic disease dyslipidemia [18], characterized by highly elevated the levels of **s**erum lipid in blood and tissues, with an increased risk for atherosclerosis and CHD that is independent of plasma cholesterol concentrations [19]. It has recently been shown that these genes play a key role in the RCT pathway and the prevention of atherosclerosis through their up regulation by liver X receptor (LXR) agonists [20,21]. The human *ABCG8* is located on chromosome 2p21. The single nucleotide polymorphism (SNP) of rs4148217, is a “C” to “A” substitution at amino acid 400 in exon 8. This SNP has been reported to influence the metabolism of plant sterols or cholesterol by intensive investigation in several Caucasian populations [22-25].

China is a multi-ethnic country with 56 ethnic groups. Han nationality is the largest ethnic group, and Mulao nationality (also known as Mulam) is one of the 55 minorities with population of 207,352 according to 2000, the fifth national census statistics. A previous study has shown that the genetic distance between Mulao nationality and other minorities in Guangxi was much closer than that between Mulao and Han or Uighur nationality [26]. In a previous study, we also found that the associations of both *GALNT2* rs2144300 and rs4846914 SNPs and serum lipid levels were different in the Mulao and Han populations [27]. We hypothesized that some genetic polymorphisms may be different between the two ethnic groups. Although several previous studies have shown the association of *ABCG8* rs4148217 SNP and CHD and/or dyslipidemia, the results are inconsistent [23,28-33]. Thus, the aim of the present study was to detect the association of *ABCG8* rs4148217 (T400K) SNP and several environmental factors with serum lipid profiles in the Mulao and Han populations.

## Materials and methods

### Study population

The study subjects included Mulao and Han nationalities. The group of Mulao nationality consisted of 634 people (range: 18–86 years). There were 267 men (42.2 %) and 367 women (57.8 %) with a mean age of 51.46 ± 15.47 years. The group of Han nationality comprised of 717 participants (range 18–86 years). There were 310 males (43.2 %) and 407 females (56.8 %) with an average age of 52.97 ± 15.04 years. All subjects were rural agricultural workers and were randomly selected from our previous stratified randomized cluster samples. All study subjects had no evidence of any chronic illness. The participants with a history of atherosclerosis, CHD and diabetes have been excluded. None of them were using lipid-lowering medication such as statins or fibrates. The study design was approved by the Ethics Committee of the First Affiliated Hospital, Guangxi Medical University. Informed consent was obtained from all subjects.

### Epidemiological survey

The survey was carried out using internationally standardized methods [34]. All participants underwent a complete history, physical examination, and laboratory assessment of cardiovascular risk factors. The alcohol information included questions about the number of liangs (about 50 g) of rice wine, corn wine, rum, beer, or liquor consumed during the preceding 12 months. Alcohol consumption was categorized into groups of grams of alcohol per day: ≤ 25 and > 25. Smoking status was categorized into groups of cigarettes per day: ≤ 20 and > 20. At the physical examination, several parameters including body height, weight, and waist circumference were measured. Sitting blood pressure was measured three times with the use of a mercury sphygmomanometer after the subjects had a 5-minute rest, and the average of the three measurements was used for the level of blood pressure. Body weight, to the nearest 50 grams, was measured using a portable balance scale. Body height was measured, to the nearest 0.5 cm, using a portable steel measuring device. From these two measurements body mass index (BMI, kg/m2) was calculated.

### Laboratory methods

Blood sample was drawn after fasting overnight. The sample was transferred to the glass tube to measure serum lipid levels and put into tubes with anticoagulate solution to extract deoxyribonucleic acid (DNA). Serum TC, TG, HDL-C, and LDL-C levels in the samples were measured by enzymatic methods with commercially available kits (RANDOX Laboratories Ltd., Ardmore, Diamond Road, Crumlin Co. Antrim, United Kingdom, BT29 4QY; Daiichi Pure Chemicals Co., Ltd., Tokyo, Japan). Serum ApoA1 and ApoB levels were detected by the immunoturbidimetric immunoassay using a commercial kit (RANDOX Laboratories Ltd.). All determinations were performed with an autoanalyzer (Type 7170A; Hitachi Ltd., Tokyo, Japan) in the Clinical Science Experiment Center of the First Affiliated Hospital, Guangxi Medical University [35,36].

### DNA amplification and genotyping

Genomic DNA was extracted from peripheral blood leukocytes by the phenol-chloroform method [37]. The *ABCG8* rs4148217 SNP was analyzed by polymerase chain reaction and restriction fragment length polymorphism (PCR-RFLP). The amplification was performed using the following forward and reverse primers: 5'-GTGCGTGACTTAGATGACTT-3' and 5'-GCGGGTTCAGTAATAAAATG-3' (Sangon, Shanghai, People’s Republic of China). The PCR mixture (25 μL of total volume) comprised 12.5 μL 2 × *Taq* PCR MasterMix (constituent: 0.1 U *Taq* polymerase/μL, 500 μM dNTP each and PCR buffer); nuclease-free water 8.5 mL; 1.0 μL of each primer (10 μmo1/L) and 100 ng (2 μL) of genomic DNA. The PCR conditions were as follows: pre-denaturation at 94°C for 5 min; followed by 35 cycles of denaturation at 95°C for 30 s, annealing at 57°C for 30 s, elongation for 45 s at 72°C and a final extension of 5 min at 72°C. The amplicons were digested by endonucleases recognizing allele-specific restriction sites with *Tru*1i. All methods were designed to include an obligate cleavage site within the amplicon to facilitate monitoring the efficacy of enzymatic digestion. Then 5 U of *Tru*1i enzyme was added directly to the PCR products (5 μL) and digested at 65°C overnight. After restriction enzyme digestion of the amplified DNA, genotypes were identified by electrophoresis on 2 % agarose gels and visualized with ethidium-bromide staining ultraviolet illumination. Genotypes were scored by an experienced reader blinded to epidemiological data and serum lipid levels. Six samples (CC, CA and AA genotypes in two; respectively) detected by the PCR-RFLP were also confirmed by direct sequencing. The PCR products were purified by low melting point gel electrophoresis and phenol extraction, and then the DNA sequences were analyzed in Shanghai Sangon Biological Engineering Technology & Services Co., Ltd., People’s Republic of China.

### Diagnostic criteria

The normal values of serum TC, TG, HDL-C, LDL-C, ApoA1, ApoB levels and the ratio of ApoA1 to ApoB in our Clinical Science Experiment Center were 3.10-5.17, 0.56-1.70, 1.16-1.42, 2.70-3.10 mmol/L, 1.20-1.60, 0.80-1.05 g/L, and 1.00-2.50; respectively. The individuals with TC > 5.17 mmol/L and/or TG > 1.70 mmol/L were defined as hyperlipidemic [35]. Hypertension was diagnosed according to the criteria of 1999 World Health Organization-International Society of Hypertension Guidelines for the management of hypertension [38]. The diagnostic criteria of overweight and obesity were according to the Cooperative Meta-analysis Group of China Obesity Task Force. Normal weight, overweight and obesity were defined as a BMI < 24, 24–28, and > 28 kg/m2; respectively [39].

### Statistical analyses

Quantitative variables are represented as mean ± standard deviation (serum TG levels are presented as medians and interquartile ranges). Qualitative variables are expressed as percentages. The difference in general characteristics between the two ethnic groups was tested by the Student’s unpaired *t*-test. Allele frequency was determined via direct counting, and the standard goodness-of-fit test was used to test the Hardy-Weinberg equilibrium. Genotype frequencies in Mulao and Han nationalities were analyzed by chi-square test. Analysis of covariance (ANCOVA) was applied to evaluate the association of genotypes and serum lipid parameters. Age, sex, BMI, blood pressure, alcohol consumption, and cigarette smoking were adjusted for the statistical analysis. In order to assess the association of serum lipid levels with genotypes (CA/AA = 0, CC = 1) and several environment factors, multiple linear regression analyses were also performed in the combined population of Mulao and Han, Mulao, Han, males, and females; respectively. The statistical software package SPSS 13.0 (SPSS Inc., Chicago, Illinois) was applied to statistical analyses. A two-tailed *P* value of less than 0.05 was considered statistically significant.

## Results

### General characteristics and serum lipid levels

The general characteristics of the two nationalities are detailed in Table 1. The levels of BMI, diastolic blood pressure and ApoA1 were lower but the levels of ApoB and the percentages of subjects who consumed alcohol were higher in Mulao than in Han (*P* < 0.05-0.001). There were no significant differences in the levels of age, height, weight, waist circumference, systolic blood pressure, pulse pressure, blood glucose; TC, TG, HDL-C, LDL-C, the ratio of ApoA1 to ApoB; the percentages of subjects who smoked cigarettes and the ratio of male to female between the two ethnic groups (*P* > 0.05 for all).

**Table 1:** Comparison of demography, lifestyle and serum lipid levels between Mulao and Han nationalities

**Parameter**	**Han**	**Mulao**	***t***** (*****x***^**2**^**)**	***P***
Number	717	634		
Male/female	310/407	267/367	0.173	0.677
Age (years)	52.97 ± 15.04	51.46 ± 15.47	1.813	0.070
Height (cm)	154.44 ± 8.04	155.18 ± 8.03	−1.678	0.094
Weight (kg)	53.60 ± 9.02	52.68 ± 9.49	1.834	0.067
Body mass index (kg/m^2^)	22.45 ± 3.39	21.82 ± 3.15	3.532	0.000
Waist circumference (cm)	75.52 ± 7.82	75.00 ± 8.62	1.166	0.244
Cigarette smoking [(n %)]				
Nonsmoker	557 (77.70)	487 (76.80)		
≤ 20 cigarettes/day	140 (19.50)	124 (19.60)		
> 20 cigarettes/day	20 (2.80)	23 (3.60)	0.776	0.678
Alcohol consumption [n (%)]				
Nondrinker	558 (77.80)	470 (74.10)		
≤ 25 g/day	74 (10.30)	58 (9.10)		
> 25 g/day	85 (11.90)	106 (16.70)	6.707	0.035
Systolic blood pressure (mmHg)	130.44 ± 18.88	128.66 ± 21.17	1.640	0.101
Diastolic blood pressure (mmHg)	82.61 ± 10.77	80.59 ± 11.38	3.366	0.001
Pulse pressure (mmHg)	47.83 ± 14.47	48.10 ± 15.93	−0.293	0.771
Blood glucose (mmol/L)	6.10 ± 1.64	5.97 ± 1.59	1.464	0.143
Total cholesterol (mmol/L)	5.06 ± 1.08	4.99 ± 1.18	1.160	0.246
Triglyceride (mmol/L)	1.07 (0.90)	1.05 (0.76)	−0.954	0.340
HDL-C (mmol/L)	1.72 ± 0.41	1.73 ± 0.45	−0.280	0.779
LDL-C (mmol/L)	2.92 ± 0.84	2.94 ± 0.87	−0.391	0.696
Apolipoprotein (Apo) A1 (g/L)	1.34 ± 0.25	1.30 ± 0.38	2.455	0.014
ApoB (g/L)	0.85 ± 0.20	0.98 ± 0.58	−4.864	0.000
ApoA1/ApoB	1.64 ± 0.49	1.61 ± 1.04	0.692	0.489

HDL-C, high-density lipoprotein cholesterol; LDL-C, low-density lipoprotein cholesterol. The value of triglyceride was presented as median (interquartile range), the difference between the two ethnic groups was determined by the Wilcoxon-Mann–Whitney test.

### Electrophoresis and genotyping

**Figure 1:**
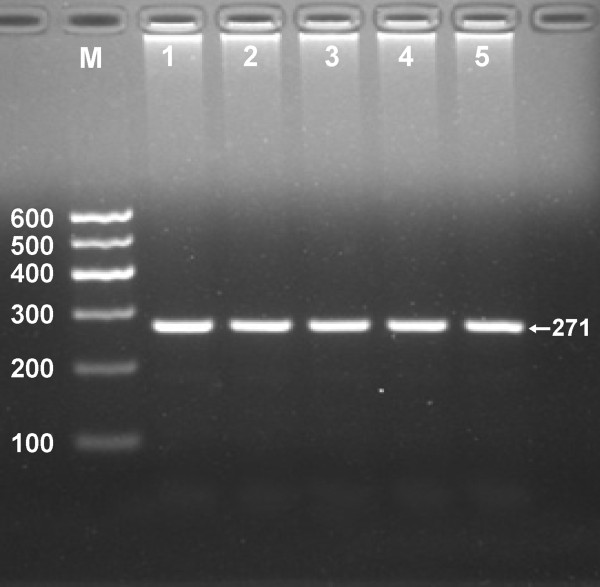
**Electrophoresis of PCR products of the samples.** Lane M, 100 bp marker ladder; lanes 1–5, samples. The 271 bp bands are the PCR products.

**Figure 2:**
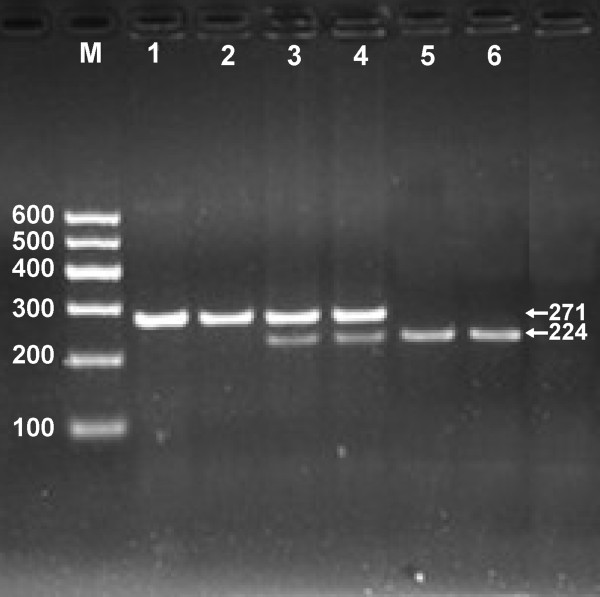
**Genotyping of the*ABCG8*rs4148217 SNP.** Lane M, 100 bp marker ladder; lanes 1 and 2, CC genotype (271 bp); lanes 3 and 4, CA genotype (271-, 224- and 47-bp); and lanes 5 and 6, AA genotype (224- and 47-bp). The 47 bp fragment was invisible in the gel owing to its fast migration speed.

The PCR products of 271 bp nucleotide sequences are shown in Figure 1. The genotypes of CC (271 bp), CA (271-, 224- and 47-bp) and AA (224- and 47-bp) are shown in Figure 2. The 47 bp fragment was invisible in the gel owing to its fast migration speed.

### Genotypic and allelic frequencies

The genotypic distribution was in Hardy-Weinberg equilibrium in both Mulao and Han nationalities. The frequencies of CC, CA and AA genotypes were 77.9 %, 20.7 % and 1.4 % in Mulao, and 84.8 %, 14.6 % and 0.6 % in Han (*P* < 0.01); respectively. The frequency of C and A alleles was 88.2 % and 11.8 % in Mulao, and 92.1 % and 7.9 % in Han (*P* < 0.01); respectively. There was no significant difference in the genotypic and allelic frequencies between males and females in both ethnic groups (Table 2).

**Table 2:** Comparison of the genotypic and allelic frequencies of ABCG8 rs4148217 SNP in Mulao and Han nationalities [n (%)]

**Group**	**n**	**Genotype CC CA AA**			**Allele C A**	
Han	717	608 (84.80)	105 (14.60)	4 (0.60)	1321 (92.10)	113 (7.90)
Mulao	634	494 (77.90)	131 (20.70)	9 (1.40)	1119 (88.20)	149 (11.80)
*x*^2^	–	11.525			11.515	
*P*	–	0.003			0.001	
Han						
Male	310	268 (86.50)	42 (13. 50)	0 (0.00)	578 (93.20)	42 (6.80)
Female	407	340 (83.50)	63 (15.50)	4 (1.00)	743 (91.30)	71 (8.70)
*x*^2^	–	3.671			1.840	
*P*	–	0.160			0.175	
Mulao						
Male	267	203 (76.00)	63 (23.60)	1 (0.40)	469 (87.80)	65 (12.20)
Female	367	291 (79.30)	68 (18.50)	8 (2.20)	650 (88.60)	84 (11.40)
*x*^2^	–	5.680			0.158	
*P*	–	0.058			0.691	

### Results of sequencing

**Figure 3:**
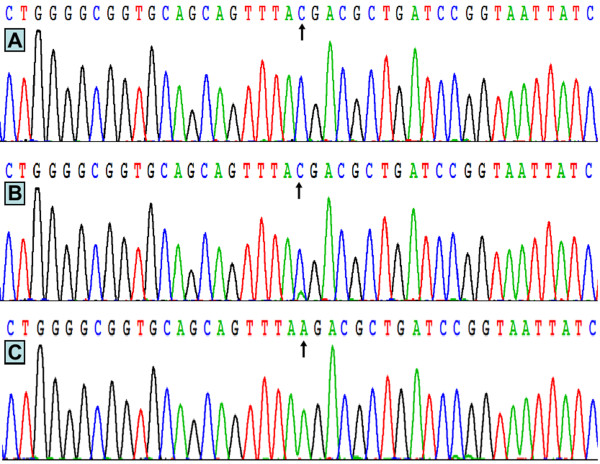
**A part of the nucleotide sequence of the*ABCG8*rs4148217 SNP.** (**A**) CC genotype; (**B**) CA genotype; (**C**) AA genotype.

The results were shown as CC, CA and AA genotypes by PCR-RFLP, the CC, CA and AA genotypes were also confirmed by sequencing (Figure 3); respectively.

### Genotypes and serum lipid levels

As revealed in Table 3, the levels of HDL-C and ApoA1 in Han were different between the CC and CA/AA genotypes (*P* < 0.05), the A allele carriers had lower HDL-C and ApoA1 levels than the A allele noncarriers. The levels of ApoA1 in Mulao were different between the CC and CA/AA genotypes (*P* < 0.05), the A allele carriers had lower ApoA1 levels than the A allele noncarriers. When serum lipid parameters were analyzed according to sex, we found that the A allele carriers in Han had lower HDL-C and higher TG levels in females but not in males than the A allele noncarriers (*P* < 0.05 for each), and the A allele carriers in Mulao had lower ApoA1 levels in females but not in males than the A allele noncarriers (*P* < 0.05).

**Table 3:** Comparison of the genotypes and serum lipid levels between Mulao and Han nationalities

**Genotype**	**n**	**TC (mmol/L)**	**TG (mmol/L)**	**HDL-C (mmol/L)**	**LDL-C (mmol/L)**	**ApoA1 (g/L)**	**ApoB (g/L)**	**ApoA1/ ApoB**
Han								
CC	608	5.08 ± 1.11	1.08(0.89)	1.73 ± 0.41	2.93 ± 0.86	1.35 ± 0.25	0.87 ± 0.20	1.64 ± 0.50
CA/AA	109	4.98 ± 0.92	1.01(0.82)	1.68 ± 0.39	2.89 ± 0.73	1.31 ± 0.24	0.85 ± 0.18	1.61 ± 0.44
*F*		0.780	−0.253	3.073	0.276	3.684	0.446	0.412
*P*		0.378	0.800	0.047	0.599	0.026	0.504	0.521
Han/male								
CC	268	5.27 ± 1.23	1.23(1.19)	1.67 ± 0.43	2.96 ± 0.90	1.36 ± 0.30	0.93 ± 0.21	1.54 ± 0.48
CA/AA	42	5.01 ± 0.57	0.95(0.75)	1.67 ± 0.40	2.82 ± 0.47	1.32 ± 0.20	0.88 ± 0.16	1.56 ± 0.44
*F*	–	1.905	−1.741	0.021	0.994	1.013	1.832	0.038
*P*	–	0.168	0.082	0.884	0.320	0.315	0.177	0.846
Han/female								
CC	340	4.92 ± 0.98	0.97(0.76)	1.78 ± 0.39	2.91 ± 0.84	1.34 ± 0.21	0.82 ± 0.19	1.72 ± 0.50
CA/AA	67	4.96 ± 1.09	1.02(0.98)	1.70 ± 0.39	2.93 ± 0.85	1.32 ± 0.26	0.84 ± 0.19	1.64 ± 0.43
*F*	–	0.077	−1.990	3.018	0.025	0.300	0.422	1.468
*P*	–	0.781	0.047	0.049	0.875	0.584	0.516	0.226
Mulao								
CC	494	4.99 ± 1.20	1.04(0.73)	1.73 ± 0.45	2.95 ± 0.86	1.35 ± 0.40	0.99 ± 0.61	1.61 ± 1.13
CA/AA	140	5.01 ± 1.15	1.07(0.93)	1.71 ± 0.47	2.93 ± 0.89	1.29 ± 0.45	0.92 ± 0.45	1.61 ± 0.61
*F*	–	0.002	−0.852	0.250	0.069	3.032	0.644	0.000
*P*	–	0.966	0.394	0.617	0.793	0.048	0.200	0.990
Mulao/male								
CC	203	5.07 ± 1.26	1.11(0.96)	1.71 ± 0.50	2.95 ± 0.83	1.31 ± 0.46	1.08 ± 0.71	1.49 ± 0.70
CA/AA	64	5.10 ± 1.07	1.25(1.38)	1.67 ± 0.47	2.95 ± 0.79	1.30 ± 0.37	1.00 ± 0.53	1.45 ± 0.60
*F*	–	0.019	−1.223	0.387	0.000	0.021	0.701	0.139
*P*	–	0.892	0.222	0.534	1.000	0.886	0.403	0.710
Mulao/female								
CC	291	4.93 ± 1.16	1.02(0.65)	1.75 ± 0.40	2.94 ± 0.88	1.36 ± 0.36	0.93 ± 0.52	1.69 ± 1.35
CA/AA	76	4.89 ± 1.21	1.01(0.68)	1.75 ± 0.46	2.90 ± 0.96	1.28 ± 0.38	0.85 ± 0.37	1.74 ± 0.59
*F*	–	0.073	−0.217	0.000	0.125	3.901	1.437	0.099
*P*	–	0. 786	0.828	0.994	0.724	0.021	0.231	0.754

TC, total cholesterol; TG, triglyceride; HDL-C, high-density lipoprotein cholesterol; LDL-C, low-density lipoprotein cholesterol; ApoA1, apolipoprotein A1; ApoB, apolipoprotein B; ApoA1/ApoB, the ratio of apolipoprotein A1 to apolipoprotein B. The values of TG were presented as median (interquartile range). The difference among the genotypes was determined by the Kruskal-Wallis test or the Wilcoxon-Mann–Whitney test.

### Risk factors for serum lipid parameters

As shown in Table 4, multiple linear regression analyses showed that the levels of ApoA1 and ApoB in Mulao but not in Han were correlated with genotypes (*P* < 0.01 for each). When the regression analysis was performed according to sex, we showed that the levels of TG and HDL-C in Han, and ApoA1 in Mulao were associated with genotypes in females but not in males (*P* < 0.05-0.01).

**Table 4:** Relationship between serum lipid parameters and genotypes in Mulao and Han nationalities

**Lipid**	**Genotype**	**Unstandardized coefficient**	**Std. error**	**Standardized coefficient**	***t***	***P***
Mulao						
ApoA1	Genotype	0.153	0.056	0.093	2.738	0.006
ApoB	Genotype	0.035	0.012	0.091	2.893	0.004
Han/female						
TG	Genotype	0.477	0.152	0.153	3.134	0.002
HDL-C	Genotype	0.314	0.092	0.183	3.433	0.001
Mulao/female						
Apo A1	Genotype	0.039	0.019	0.076	2.068	0.039

TC, total cholesterol; HDL-C, high-density lipoprotein cholesterol; LDL-C, low-density lipoprotein cholesterol; ApoA1, apolipoprotein A1; ApoB, apolipoprotein B; ApoA1/ApoB, the ratio of apolipoprotein A1 to apolipoprotein B.

Serum lipid parameters were also correlated with several environment factors such as sex, age, BMI, alcohol consumption, cigarette smoking, and blood pressure in both ethnic groups (*P* < 0.05-0.001; Tables 5 and 6).

**Table 5:** Relationship between serum lipid parameters and environmental risk factors in Mulao and Han nationalities

**Lipid**	**Risk factor**	**Unstandardized coefficient**	**Std. error**	**Standardized coefficient**	***t***	***P***
Han plus Mulao						
TC	Age	0.011	0.002	0.147	5.475	0.000
	Alcohol consumption	0.212	0.043	0.132	4.976	0.000
	Diastolic blood pressure	0.012	0.003	0.116	4.194	0.001
	Waist circumference	0.011	0.002	0.147	5.475	0.000
TG	Waist circumference	0.052	0.007	0.209	7.773	0.000
	Diastolic blood pressure	0.019	0.005	0.101	3.793	0.000
	Blood glucose	0.115	0.033	0.091	3.528	0.000
	Alcohol consumption	0.442	0.076	0.145	5,561	0.006
HDL-C	Waist circumference	−0.007	0.002	−0.141	−3.844	0.000
	Alcohol consumption	0.106	0.019	0.173	5.732	0.000
	Body mass index	−0.022	0.005	−0.116	−4.497	0.000
	Age	0.002	0.001	0.055	2.065	0.039
	Gender	0.107	0.028	0.123	3.825	0.000
LDL-C	Age	0.010	0.001	0.183	6.952	0.000
	Body mass index	0.047	0.007	0.181	6.860	0.000
ApoA1	Age	0.001	0.001	0.062	2.327	0.020
	Gender	0.073	0.020	0.113	3.825	0.000
	Body mass index	−0.009	0.003	−0.093	−3.473	0.001
	Alcohol consumption	0.126	0.014	0.276	8.515	0.000
	Ethnic group	−0.047	0.017	0.073	−2.327	0.020
ApoB	Waist circumference	0.009	0.001	0.179	6.787	0.000
	Ethnic group	−0.123	0.022	−0.145	5.258	0.000
	Blood glucose	0.020	0.007	0.074	2.816	0.005
	Systolic blood pressure	0.002	0.001	0.088	3.279	0.001
	Gender	0.072	0.023	0.084	3.099	0.002
ApoA1/ApoB	Waist circumference	−0.011	0.004	−0.117	−3.129	0.002
	Gender	0.209	0.052	0.130	4.008	0.000
	Alcohol consumption	0.102	0.036	0.090	2.832	0.005
	Body mass index	−0.023	0.009	−0.097	−2.649	0.008
	Age	−0.004	0.001	−0.084	−3.182	0.001
Han						
TC	Waist circumference	0.016	0.005	0.113	3.065	0.002
	Age	0.009	0.003	0.119	3.176	0.002
	Alcohol consumption	0.269	0.056	0.172	4.808	0.000
	Diastolic blood pressure	0.019	0.004	0.187	5.002	0.000
	Blood glucose	0.055	0.024	0.083	2.290	0.022
TG	Wrist circumference	0.084	0.014	0.275	5.850	0.000
	Blood glucose	0.246	0.052	0.169	4.689	0.000
	Diastolic blood pressure	0.036	0.008	0.162	4.380	0.000
	Age	−0.014	0.006	−0.087	−2.341	0.020
	Body mass index	0.087	0.033	0.123	−2.657	0.008
	Alcohol consumption	0.559	0.122	0.162	4.574	0.000
HDL-C	Waist circumference	−0.012	0.003	−0.224	−4.699	0.000
	Gender	0.167	0.039	0.201	4.272	0.000
	Alcohol consumption	0.114	0.025	0.192	4.552	0.000
	Body mass index	−0.012	0.006	−0.102	−2.184	0.029
	Cigarette smoking	0.085	0.035	0.101	2.406	0.016
LDL-C	Age	0.012	0.002	0.207	5.722	0.000
	Body mass index	0.048	0.009	0.194	5.373	0.000
ApoA1	Alcohol consumption	0.133	0.015	0.372	8.975	0.000
	Body mass index	−0.014	0.003	−0.195	−5.539	0.000
	Gender	0.103	0.023	0.206	4.522	0.000
	Cigarette smoking	0.082	0.021	0.160	3.885	0.000
ApoB	Waist circumference	0.005	0.001	0.191	4.177	0.000
	Gender	−0.040	0.016	−0.098	−2.417	0.016
	Ages	0.001	0.001	0.097	2.548	0.011
	Systolic blood pressure	0.003	0.001	0.193	3.915	0.000
	Blood glucose	0.020	0.004	0.167	4.859	0.000
	Alcohol consumption	0.032	0.011	0.111	2.822	0.005
	Body mass index	0.008	0.003	0.135	3.032	0.003
ApoA1/ApoB	Waist circumference	−0.010	0.003	−0.161	−3.554	0.000
	Body mass index	−0.030	0.006	−0.210	−4.650	0.000
	Age	−0.003	0.001	−0.1101	−2.957	0.003
	Alcohol consumption	0.111	0.029	0.157	3.856	0.000
	Cigarette smoking	0.167	0.041	0.165	4.097	0.000
	Gender	0.253	0.046	0.248	5.400	0.000
Mulao						
TC	Age	0.012	0.003	0.161	4.170	0.000
	Alcohol consumption	0.171	0.065	0.102	2.621	0.009
	Waist circumference	−0.020	0.005	−0.161	4.170	0.000
TG	Waist circumference	0.045	0.007	0.252	6.567	0.000
	Alcohol consumption	0.278	0.083	0.128	3.342	0.001
HDL-C	Alcohol consumption	0.088	0.029	0.139	3.021	0.003
	Gender	0.093	0.042	0.102	2.218	0.027
	Body mass i ndex	0.040	0.005	−0.280	−7.339	0.000
LDL-C	Age	0.008	0.002	0.147	4.042	0.000
	Body mass index	0.049	0.010	0.177	4.599	0.000
ApoA1	Alcohol consumption	0.112	0.026	0.206	4.350	0.000
	Gender	0.082	0.037	0.105	2.221	0.027
ApoB	Waist circumference	0.011	0.003	0.168	4.190	0.000
	Gender	−0.113	0.046	−0.097	−2.439	0.015
ApoA1/ApoB	Age	−0.006	0.003	−0.086	−2.179	0.028
	Waist circumference	−0.018	0.005	0.152	−3.872	0.000
	Cigarette smoking	−0.159	0.078	−0.080	−2.031	0.043

TC, total cholesterol; TG, triglyceride; HDL-C, high-density lipoprotein cholesterol; LDL-C, low-density lipoprotein cholesterol; ApoA1, apolipoprotein A1; ApoB, apolipoprotein B; ApoA1/ApoB, the ratio of apolipoprotein A1 to apolipoprotein B.

**Table 6:** Relationship between serum lipid parameters and and environmental risk factors in males and females in both ethnic groups

**Lipid**	**Risk factor**	**Unstandardized coefficient**	**Std. error**	**Standardized coefficient**	***t***	***P***
Han/male						
TC	Diastolic blood pressure	0.033	0.005	0.322	6.013	0.000
	Alcohol consumption	0.289	0.073	0.209	3.980	0.000
	Blood glucose	0.083	0.036	0.122	2.320	0.021
TG	Waist circumference	0.080	0.023	0.189	3.473	0.000
	Cigarette smoking	1,240	0.286	0.231	4.376	0.000
	Blood glucose	0.439	0.107	0.228	4.108	0.000
	Age	−0.024	0.012	−0.118	−2.082	0.038
HDL-C	Waist circumference	−0.014	0.003	−0.252	−4.650	0.000
	Alcohol consumption	0.107	0.027	0.210	3.883	0.000
LDL-C	Cigarette smoking	−0.329	0.074	−0.238	−4.436	0.000
	Body mass index	0.039	0.012	0.172	3.216	0.001
ApoA1	Alcohol consumption	0.133	0.018	0.393	7.565	0.000
	Body mass index	−0.013	0.004	−0.166	−3.269	0.001
	Cigarette smoking	0.067	0.024	0.144	2.756	0.006
ApoB	Body mass index	0.006	0.003	0.118	1.980	0.049
	Blood glucose	0.022	0.006	0.187	3.736	0.000
	Alcohol consumption	0.035	0.012	0.143	2.851	0.005
	Diastolic blood pressure	0.004	0.001	0.238	4.644	0.000
	Waist circumference	0.005	0.002	0.176	2.942	0.004
ApoA1/ApoB	Body mass index	−0.029	0.008	−0.230	−3.742	0.000
	Cigarette smoking	0.104	0.041	0.136	2.554	0.011
	Alcohol consumption	0.104	0.029	0.187	3.528	0.000
	Waist circumference	−0.011	0.004	0.183	−2.996	0.003
Han/female						
TC	Age	0.022	0.003	0.315	7.271	0.000
	Waist circumference	0.023	0.006	0.161	3.720	0.000
TG	Waist circumference	0.040	0.006	0.277	6.317	0.000
	Diastolic blood pressure	0.011	0.004	0.110	2.512	0.012
	Blood glucose	0.128	0.029	0.187	4.369	0.000
HDL-C	Waist circumference	−0.008	0.004	−0.102	−2.227	0.026
LDL-C	Age	0.020	0.003	0.336	7.585	0.000
	Waist circumference	0.021	0.005	0.175	4.031	0.000
	Cigarette smoking	−0.527	0.244	−0.095	−2.163	0.031
ApoA1	Cigarette smoking	0.155	0.072	0.099	2.152	0.032
	Waist circumference	0.023	0.006	0.161	3.720	0.000
ApoB	Blood glucose	0.019	0.006	0.151	3.466	0.001
	Age	0.003	0.001	0.195	4.287	0.000
	Cigarette smoking	−0.118	0.053	−0.097	−2.238	0.026
	Waist circumference	0.007	0.001	0.273	6.379	0.000
ApoA1/ApoB	Body mass index	−0.038	0.007	−0.223	−5.127	0.000
	Cigarette smoking	0.590	0.143	0.183	4.114	0.000
	Age	−0.007	0.002	−0.219	−4.903	0.000
Mulao/male						
TC	Body mass index	0.064	0.021	0.173	3.117	0.002
	Alcohol consumption	0.169	0.071	0.133	2.385	0.018
TG	Waist circumference	0.062	0.012	0.276	5.053	0.000
HDL-C	Alcohol consumption	0.106	0.029	0.194	3.602	0.000
	Body mass index	−0.045	0.009	−0.284	−5.276	0.000
LDL-C	Body mass index	0.041	0.014	0.163	2.901	0.004
ApoA1	Alcohol consumption	0.118	0.025	0.258	4.697	0.000
ApoB	Waist circumference	0.011	0.004	0.156	2.785	0.006
ApoA1/ApoB	Alcohol consumption	0.116	0.042	0.150	2.736	0.007
	Waist circumference	−0.018	0.004	−0.230	−4.184	0.000
Mulao/female						
TC	Age	0.014	0.004	0.181	3.726	0.000
	Body mass index	0.046	0.019	0.120	2.465	0.014
TG	Body mass index	0.062	0.013	0.233	4.822	0.000
HDL-C	Body mass index	−0.035	0.007	−0.251	−5.219	0.000
LDL-C	Body mass index	0.053	0.014	0.183	3.845	0.000
	Age	0.013	0.003	0.218	4.577	0.000
ApoA1	Waist circumference	−0.018	0.004	−0.230	−4.184	0.000
	Body mass index	0.046	0.019	0.120	2.465	0.014
	Cigarette smoking	−0.118	0.055	−0.100	−3.238	0.006
ApoB	Blood glucose	0.046	0.017	0.128	2.638	0.009
	Wrist circumference	0.013	0.003	0.205	4.228	0.000
ApoA1/ApoB	Waist circumference	−0.016	0.008	−0.128	−2.807	0.018
	Age	−0.011	0.004	−0.140	−2.849	0.005

TC, total cholesterol; TG, triglyceride; HDL-C, high-density lipoprotein cholesterol; LDL-C, low-density lipoprotein cholesterol; ApoA1, apolipoprotein A1; ApoB, apolipoprotein B; ApoA1/ApoB, the ratio of apolipoprotein A1 to apolipoprotein B.

## Discussion

The present study shows that the levels of ApoA1 were lower and the levels of ApoB were higher in Mulao than in Han. There was no significant difference in the levels of TC, TG, HDL-C, LDL-C and the ratio of ApoA1 to ApoB between the two ethnic groups. It is well known that dyslipidemia is a complex trait caused by multiple environmental and genetic factors and their interactions. Mulao nationality is an isolated minority in China. They have their life habits and intra-ethnic marriage customs. There was a preference for marriage to mother’s brother’s daughter. Therefore, Mulao nationality has a homogeneous genetic background which may be different from that in Han nationality.

In the present study, we showed that the genotypic distribution of *ABCG8* rs4148217 SNP was different between the two ethnic groups. The frequency of CC genotype was lower in Mulao (77.9 %) than in Han (84.8 %). The frequency of C allele was also lower in Mulao (88.2 %) than in Han (92.1 %, *P* < 0.05). The genotypic and allelic frequencies of *ABCG8* rs4148217 SNP in different populations are inconsistent. Several similar researches have carried out in European and North American populations. In a study in Hungary Caucasian race, Szilvási *et al*. [40] reported that the frequency of CC genotype in CHD patients, stroke patients, and controls were 63.5 %, 68.0 %, and 64.9 %; respectively. The another study in the Boston Puerto Rican Health Study in American, Junyent *et al*. [41] determined that the frequencies of CC and CA/AA genotypes were 60.0 % and 40.0 %; respectively. In a study of dyslipidemic patients who were recruited from 31 community- and university-based research centers in the USA showed that the frequencies of CC and CA/AA genotypes were 58.5 % and 41.50 %; respectively [42]. The Netherlands’ study showed that the frequency of CC and CA/AA genotypes were 68.7 % and 31.30 %; respectively [43]. The Germany in siblings with gallstones’ research showed that the frequency of CC and CA/AA genotypes were 58.3 % and 41.7 %; respectively [31]. The frequencies of CC, CA and AA genotypes in the Czech population were 65.4 %, 31.3 %, and 3.3 %; respectively [23]. In a study of patients with gallstone in Chinese Shanghai, Wang *et al*. [33] showed that the frequency of CC, CA and AA genotypes were 83.1 %, 16.4 %, and 0.5 %; respectively, which was similar to our results of the Han population. These results suggest that the *ABCG8* rs4148217 SNP may have a racial/ethnic specificity.

The association of *ABCG8* rs4148217 SNP and serum lipid levels is different or contradictory in different ethnic groups. In the Boston Puerto Rican Health Study, Junyent *et al*. [44] showed that low HDL-C concentrations were observed in CC genotype of *ABCG8* rs4148217 SNP (*P* = 0.012). The research of Shanghai populations in China showed that males with the A allele had lower plasma TG (*P* = 0.044) than CC homozygotes, but no such association was found in female [33]. In the Netherlands populations, Plat *et al*. [43] showed that cholesterol-standardized serum campesterol and sitosterol concentrations were significantly associated with the *ABCG8* rs4148217 genotypes, as were changes in serum plant sterol concentrations after consumption of plant stanols; the reduction of cholesterol for sitosterol in CC subjects was significantly greater compared with the subjects with the CA genotype (*P* = 0.021) and the subjects with the AA genotype (*P* = 0.047). No association with serum LDL-C was found. The Germany in siblings with gallstones study showed that male CC homozygotes exhibited a greater decrease TC (*P* < 0.02) and LDL-C (*P* < 0.04) than CA/AA carriers after dietary changes. No such association was observed in females [31]. Zhao *et al*. [45] reported that the CC carriers of *ABCG8* rs4148217 SNP presented higher plasma concentrations of campesterol, sitosterol and sum of campesterol and sitosterol, respectively, as compared with CA/AA carriers. Interestingly, our data showed that the A allele carriers in Han had lower HDL-C and higher TG levels in females but not in males than the A allele noncarriers, and the A allele carriers in Mulao had lower ApoA1 levels in females but not in males than the A allele noncarriers. The levels of TG and HDL-C in Han, and ApoA1 in Mulao were associated with genotypes in females but not in males. These findings suggest that the association of *ABCG8* rs4148217 SNP and serum lipid levels is different between the Mulao and Han populations. There is a sex (female)-specific association in both ethnic groups.

The reason for above conflicting results is not fully understood, probably because of differences in study designs, sample size, pharmaceutical treatments, the methods used to determine serum lipid levels and the polymorphism or different racial/ethnic groups have different genetic background. In addtion, environmental factors were also strongly related with serum lipid levels [46]. In the present study, we showed that serum lipid parameters were correlated with age, sex, alcohol consumption, cigarette smoking, BMI, and blood pressure in both ethnic groups. These findings suggest that the environmental factors play an important role in determining serum lipid levels in these populations. For example, heavy smokers have, on average, 9 % lower HDL-C levels than matched nonsmokers [47]. Obesity is one of the most important factors in reducing HDL-C levels [48]. These factors could explain why the association between HDL-C concentration and the *ABCG8* rs4148217 SNP in diverse racial/ethnic groups was different in several previous studies.

Although Mulao and Han nationalities reside in the same region, the diet and lifestyle were different between the two ethnic groups. The people of Mulao nationality like to eat cold foods along with acidic and spicy dishes and they also like to eat animal offals which contain abundant saturated fatty acid. Long-term high-fat diets, dyslipidemia more tends to happen. Yu-Poth *et al*. [49] showed positive correlations between changes in dietary total and saturated fatty acids and changes in TC, LDL-C and HDL-C, for every 1 % decrease in energy consumed as dietary saturated fatty acid, TC decreased by 0.056 mmol/L and LDL-C by 0.05 mmol/L. Moreover, for every 1-kg decrease in body weight, TG decreased by 0.011 mmol/L and HDL-C increased by 0.011 mmol/L. Another research also showed that the effects of alcohol intake on serum lipid levels appear to change by specific individual types, patterns of alcohol intake, gender, or genotypic distribution of some SNPs [50]. Onat *et al*. [51] showed that alcohol consumption was positively associated with TG, LDL-C and ApoB in men, and negatively correlated with TG and/or not correlated with LDL-C and ApoB in women.

## Conclusion

The present study shows that the *ABCG8* rs4148217 SNP is associated with serum TG, HDL-C and ApoA1 levels in the Mulao and Han populations, but the genotypic and allelic frequencies of *ABCG8* rs4148217 SNP and the association of this SNP and serum lipid parameters are different between the two nationalities. A sex (female)-specific association is also observed in the both ethnic groups.

## Competing interests

The authors declare that they have no competing interests.

## Authors’ contributions

QL and XLW participated in the design, undertook genotyping, performed the statistical analyses, and drafted the manuscript. RXY conceived the study, participated in the design, carried out the epidemiological survey, collected the samples, and helped to draft the manuscript. All authors read and approved the final manuscript.
